# Development of a patient-oriented navigation model for patients with lung cancer and stroke in Germany

**DOI:** 10.1186/s12913-022-08063-6

**Published:** 2022-06-16

**Authors:** Kathrin Gödde, Hella Fügemann, Anke Desch, Judith Stumm, Daniel Schindel, Nina Rieckmann, Andreas Meisel, Jacqueline Müller-Nordhorn, Ute Goerling, Christine Holmberg

**Affiliations:** 1 Institute of Public Health, Charité-Universitätsmedizin Berlin, Corporate Member of Freie Universität Berlin, Humboldt-Universität zu Berlin, and Berlin Institute of Health, Berlin, Germany; 2 Institute of Social Medicine and Epidemiology, Brandenburg Medical School Theodor Fontane, Brandenburg an der Havel, Germany; 3 Institute of General Practice, Charité - Universitätsmedizin Berlin, Corporate Member of Freie Universität Berlin, Humboldt-Universität zu Berlin, and Berlin Institute of Health, Berlin, Germany; 4 Institute of Medical Sociology and Rehabilitation Science, Charité - Universitätsmedizin Berlin, Corporate Member of Freie Universität Berlin, Humboldt-Universität zu Berlin, and Berlin Institute of Health, Berlin, Germany; 5 Center for Stroke Research (CSB), Charité-Universitätsmedizin Berlin, Corporate Member of Freie Universität Berlin, Humboldt-Universität zu Berlin, and Berlin Institute of Health, Berlin, Germany; 6 Bavarian Health and Food Safety Authority, Bavarian Cancer Registry, Erlangen, Germany; 7 Charité Comprehensive Cancer Center, Charité-Universitätsmedizin Berlin, Corporate Member of Freie Universität Berlin, Humboldt-Universität zu Berlin, and Berlin Institute of Health, Berlin, Germany; 8 Faculty of Health Sciences, Brandenburg Medical School Theodor Fontane, Neuruppin, Germany

**Keywords:** Patient navigation, Lung cancer, Stroke, Barriers to care, Patient-oriented care

## Abstract

**Background:**

The concept of patient navigation was first established in the USA to support vulnerable patient groups in receiving timely and comprehensive access to cancer care. It has recently gained increasing interest in Germany to support patients with chronic diseases in a fragmented healthcare system. The aim of this paper is to present the development of such a model adapted to the German context based on the results of mixed-methods studies investigating the need for and barriers to patient-oriented care.

**Methods:**

In a process adapted from Delphi rounds, we conducted regular structured workshops with investigators of the project to discuss results of their studies and identify content and structure of the model based on the data. Workshop discussions were structured along seven core components of a navigation model including target patient groups, navigator tasks, occupational background and education of navigators, and patient-navigator interaction mode.

**Results:**

Using an approach based on empirical data of current care practices with special focus on patients’ perspectives, we developed a patient-oriented navigation model for patients who have experienced stroke and lung cancer in the German healthcare context. Patients without personal social support were viewed as struggling most with the healthcare system, as well as multimorbid and elderly patients. Navigators should serve as a longer-term contact person with a flexible contact mode and timing based on the individual situation and preferences of patients. Navigator tasks include the provision of administrative and organizational support as well as referral and guidance to available resources and beneficial health programs with special forms of knowledge. Implementation of the navigator should be flexibly located to ensure a reliable outreach to vulnerable patients for first contact in settings like specialized in-patient and out-patient settings, while navigation itself focuses on care coordination in the out-patient setting.

**Conclusion:**

Flexibility of navigator tasks needed to be a core characteristic of a navigation model to be perceived as supportive from patients’ perspectives. In a subsequent feasibility study, an intervention based on the model will be evaluated according to its acceptance, demand, and practicality.

## Introduction

The German healthcare system offers universal healthcare coverage. However, barriers to timely and patient-oriented care can occur along patients’ care paths due to a segmented organization of the system, in which in-patient and out-patient care are organized independently [1–3], and a lack of a gate-keeping system for out-patient care [4]. In addition, there is a plethora of supporting services for patients such as social care, self-help groups, athletic programs for patients and others. Acquiring information about these services can be difficult for patients and their care providers [5–8]. Even General Practitioners (GPs) as coordinators of care for old and vulnerable patients are often not aware of existing programs and services by social institutions in their city district [9]. In an aging population with an estimated increase of the population over 60 years of age from 28% (about 23.2 million people) in 2018 to 36.7% (about 28.5 million people) in 2050 in Germany [10], the pressure on the healthcare system due to healthcare for chronic disease is increasing. Hence, there is a need to optimize access to and use of existing services and resources.

Patient navigation is a concept to facilitate access to existing care services established and initially implemented in the United States as a community-based service delivery intervention in cancer care. It aimed to overcome barriers to timely cancer screening, diagnosis, and treatment for people with lower income [11–13]. The National Cancer Institute Patient Navigation Research Program described four important stages of patient navigation that are based on the care management or case management model [14, 15]. These stages involve 1) the identification of patients in need for a navigation assistance, 2) the identification of barriers of an individual patient to receive care, 3) the development of a plan to address and dismantle these barriers, and 4) the follow-up until resolution of the identified problems and barriers [15]. International studies have shown that patient navigation can influence timely access to care [16, 17], patient self-efficacy [18] and satisfaction with care [19]. High rates of satisfaction with patient navigation were reported in cancer care [20]. Navigation has also been reported to be cost-effective by reducing medical costs and hospitalizations [21]. However, results are still inconclusive as another study from the US has shown no effect of navigation on satisfaction with care [22]. In Germany, patient navigation is gaining increasing attention and is currently under discussion to be implemented as a covered healthcare service [23]. Various interventions for navigation tailored to the care of different patient populations and diseases are currently being evaluated [24, 25]. These interventions differ in terms of the diseases and patient groups they are targeting, the identification and outreach to patients in potential need, the intensity of the intervention, navigator tasks, services, roles and professional background, as well as the overall aims of the patient navigation. Mostly, studies evaluating navigation programs focus on either timely access to care services or on guideline-oriented care delivery.

In the present study we investigated lung cancer and stroke as prototypes of age-associated and chronic diseases. Both have very different and complex patient trajectories and needs for which patient navigation might potentially be appropriate. In lung cancer, the 5-year-survival rate in Germany is low with about 15% for men and 20% for women [26] and need for palliative care is often indicated early or directly after diagnosis [27]. The mortality rate after a stroke is about 50% after 5 years, and 40% of patients are permanently affected by disabilities [28]. Dependent on the severity of the acute stroke and comorbidities, intensive in-patient care is required that is often followed by a phase of in-patient and out-patient rehabilitation. Patients often face long-term changes in every-day life with need for support [29, 30]. Studies have shown deficits in risk factor management, stroke after-care and secondary prevention after stroke in Germany [31–33]. For lung cancer, a risk for potential undertreatment has been reported for elderly patients [34]. Both diseases are characterized by often complex, but very different care situations that can require the utilization and coordination of multiple healthcare providers from different disciplines and varying care settings. Such complex care situations may be overwhelming for patients as well as caregivers and difficult to coordinate for all parties, particularly as most healthcare institutions follow their routines rather than accommodate patients’ needs [23, 35, 36]. Our collection of regional support services for both diseases [7] indicated that in our study region, Berlin, a variety of different services and resources is available to cover questions that may arise for patients during different disease trajectories, but expert interviews showed that knowledge about these services is often lacking in patients as well as care providers [7].

The aim of the present study was to develop a navigation model for patients with chronic disease that is particularly patient-oriented and that fits the frame of the German healthcare system. The model focussed on lung cancer and stroke as two prototypic age-associated diseases. In line with the Institute of Medicine’s 2001 definition of patient-centered care, we aimed to develop a model that is “respectful of, and responsive to, individual patient preferences, needs and values, and ensuring that patient values guide all clinical decisions” [37].

## Materials and methods

We developed a patient navigation model within the context of a consortium for patient-oriented health services research [38], which is funded by the German Federal Ministry of Education and Research. In a process adapted from Delphi rounds [39], we conducted regular structured workshops with investigators of the project to discuss results of their studies and identify content and structure of the model based on the data. Workshop discussions were structured along seven core components of a navigation model that were identified in the literature [13, 14]. These components are displayed in Table 1. We used a data-driven approach for the model development as group discussions based upon a series of underlying studies in which we assessed barriers to optimal care as well as resources of support from the perspectives of patients, care experts, and providers [7, 40–42] (Fügemann et al., in preparation). In addition, we assessed patient’s expectations about what a navigation assistance is and what a navigator should do [43].

**Table 1:** Predefined core components to be defined in the process of development of the patient navigation model

No	Core Component
1	Target patient groups and identification strategy (at what point in the care continuum and how patients are approached?)
2	Roles and tasks of patient navigators
3	Professional background and education of navigators
4	Disease-specific versus disease-transcending components
5	Mode of patient-navigator interaction (setting, time, length of and interval between encounters)
6	Affiliation/integration of patient navigators into the healthcare system
7	Stakeholder involvement

### Underlying data

**Fig. 1:**
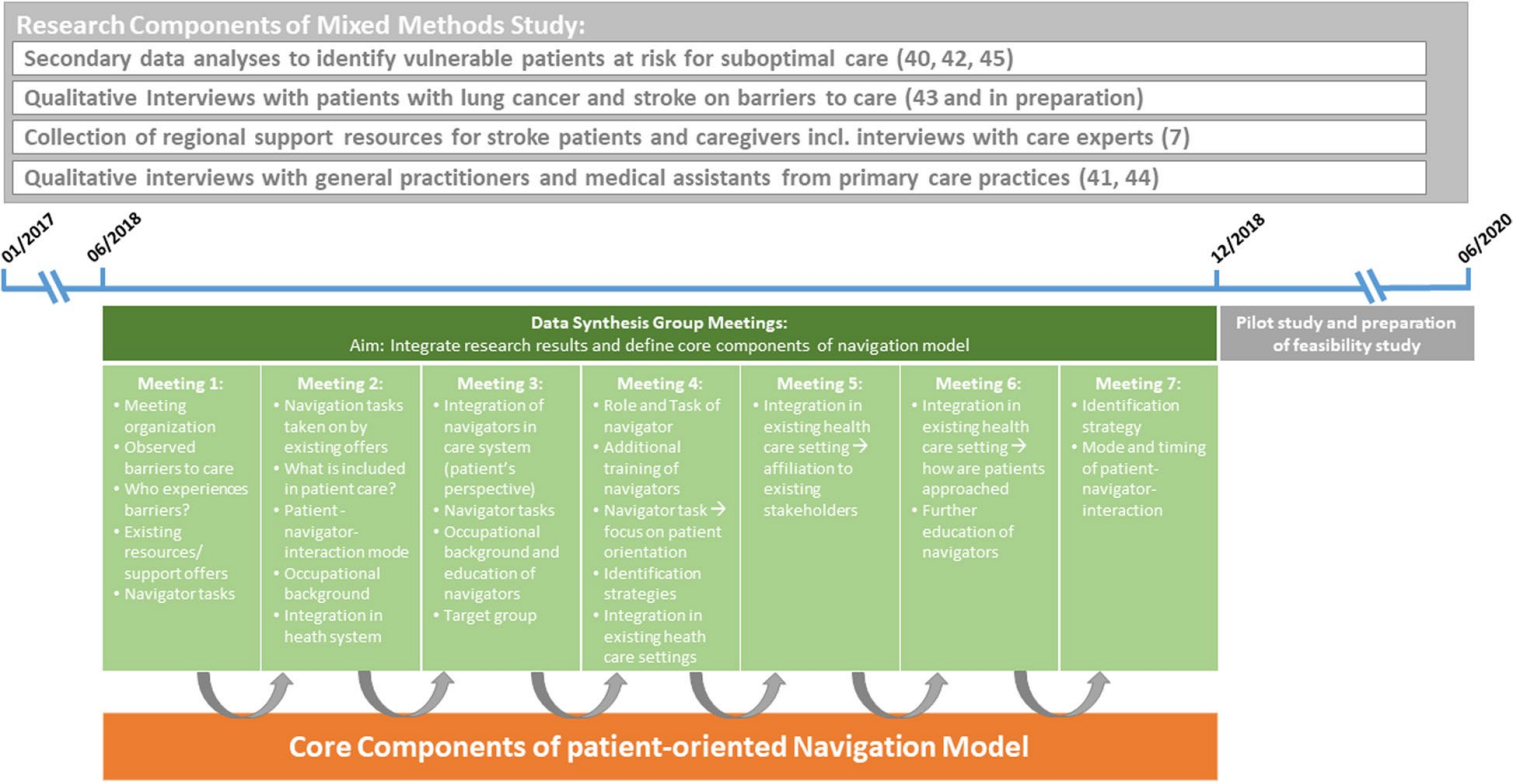
Temporal course of navigation model development. Illustrated are the different research projects conducted and considered in the development. Seven data integration meetings with different topics were conducted. After model development, a pilot phase was conducted to investigate patient accessibility and a feasibility study for the evaluation of the navigation intervention was planned

The Delphi-adapted discussions based on results and data from different research components from two projects within the research consortium were integrated in the model development (Fig. 1).

Both projects conducted mixed-methods studies investigating barriers to care from different perspectives including patients, physicians, medical assistants, and other health care workers. One project focused on the investigation of barriers along the care trajectory of patients with age-associated diseases, namely stroke and lung cancer. The second project investigated the potential of coordinating medical professions for sustainable support of multimorbid patients in the context of general practitioner practices. Results of these underlying studies present the base of the structured group discussions for the development of a patient-navigation model and are published in separate publications [7, 40–42, 44, 45].

To address open discussion points from patients’ perspective, we discussed further data from a longitudinal qualitative study with people with lung cancer (*n* = 20) or stroke (*n* = 20) that we conducted to collect a broad spectrum of patient needs and preferences at different disease trajectories. Study participants were invited to take part in three in-depth face-to-face interviews over a period of six to 12 months. The baseline and first follow-up interviews focused on expectations, suggestions, and support needs regarding patient navigation. In the second follow-up interview the developed navigation model was presented to study participants for final evaluation. For the analysis of the data, we used a descriptive qualitative approach [46–48]. After we identified all answers to the questions of the interview guide (what a navigation is and what a navigator should do from their point of view, how and where interactions with navigators should take place, who is in need of navigation), we then coded the data corpus according to deductive codes: patients´ definition of navigators, tasks of navigators, communication and interaction modes, and target groups [13, 14]. The data were used to fill the core components 1, 2, 5 and 6 of our navigation model (see Table 1) with content based on patients' expectations and ideas. Core component 7 (stakeholder involvement) presents results of the final evaluation of our model from the patients` perspective.

Further details of the interview study are described elsewhere [43]. This study was approved by Ethics committee of the Charité-Universitätsmedizin Berlin.

All methods were performed in accordance with the relevant guidelines and regulations.

### Model development process

For the adapted Delphi process we held regular structured group discussions led by the principal investigator (CH) alongside the researchers of both projects (CH; HF; UG; AD; JS; KG). In total, six researchers with different disciplinary backgrounds and expertise in qualitative and quantitative research as well as in-patient care took part in the discussions as well as a study nurse involved in study recruitment. The backgrounds of specialists included social medicine, epidemiology, public health, psychology, anthropology, life science, physiotherapy and communication science. Seven meetings were held in monthly intervals over the course of the second half of the year 2018. Meetings began 1.5 years after data collection started in the projects to ensure that analysis was already ongoing in the research components of the mixed-methods studies and results and experiences could be used for the model development (see Fig. 1). Detailed protocols were prepared for each meeting. Meetings were structured so that each of the seven predefined core component of the navigation model (Table 1) was discussed in relation to the on-going analysis of the research components of the mixed-methods studies. The initial two meetings mainly focused on the presentation of results. Subsequently, the focus was on integrating the various results and transferring them to possible models of care. Content for the core components of the navigation model was compiled by contrasting study results and finding commonalities between the results and data or by filling gaps using the different study components. In a third step, the discussion group focused on discussing possible ways to integrate the model in the German healthcare system. Finally, the model was presented to patients during qualitative interviews and to the wider research community for final adjustments and refinements. Of note, some core components were recurrently discussed when open questions remained, and a different focus of discussion was considered necessary.

## Results

The study results indicated that care needs arise in the out-patient setting and when social care was not available during the hospital stay. Also a high degree of guideline-conform treatment was seen for patients with lung cancer in the acute setting [40]. Thus, as a first consensus, we focused the navigation on the out-patient setting, as the hospital social service should be available by default to patients in the in-patient setting in Germany, including a mandatory discharge management. The navigation should, however, work very closely with this hospital service. In general, it was agreed that navigation should not interfere with or take over the tasks of existing programs or services and competencies along the care trajectory. Rather it should ensure that such services are made known and it should assist patients and care providers in accessing and using these services by referral. Their task is to build up a comprehensive network of existing providers that are incorporated along the care trajectory of a patient that may also function to provide flexible points of entry for patients with needs. This also led us to a second consensus that the navigation model should be situated somewhere in the care trajectory “where patients are” in order to enable easy access to the navigation service without adding another layer of complexity and players to the care trajectory.

In the following, we will now describe the results of the core components (Table 1) of the navigation model derived from the data integration discussions.

### Target patient groups and identification strategy

Different characteristics (“vulnerabilities”) which might increase the risk of facing barriers during a care path were identified. These were derived from interviews with patients and caregivers indicating that a lack of support from family members or other social support and multimorbidity that requires particularly complex care are such vulnerabilities.

Further target groups mentioned by patients during interviews are elderly patients, patients with physical and cognitive disabilities, patients who did not get in contact with a hospital’s social service during their stay, as well as patients who do not have a general practitioner. Furthermore, the navigation support should also be provided to caregivers of patients, as these are often closely involved in the organization of care.

“But there are also people in need of help or patients who are worse off, right? Who can no longer walk properly or only with this rollator or crutches or wheelchair. That they also get support. Once a month or so, the [navigator] checks if everything is OK." (Stroke, m, 56 years, diagnosis three months ago)

According to these observations, group discussions led to the consensus that patients should be identified and contacted in a proactive approach. In addition, pre-screening should be performed to evaluate social support e.g. via questionnaires about a patient’s social situation and support needs. Identifying vulnerable patients is a task that requires not only an active approach but also close collaboration between navigators and providers in the particular healthcare setting where patients are being approached for this service (e.g., a hospital, an outpatient clinic or a rehabilitation centre).

### Roles and tasks of patient navigators

A variety of specific tasks for a navigator aiming to reduce observed barriers to care were collected during the discussion meetings by special consideration of results from patient interviews, including: help with bureaucratic and administrative problems occurring as a result of the new disease-related living situation, support with legal forms, consultations on legal rights, education about entitlements in healthcare, social care and financial support, organization of appointments with doctors and therapists of different disciplines, organization of transportation, provision of psychologic and emotional support. From these tasks, three main tasks were derived: (1) Navigators should provide support for patients regarding bureaucratic and administrative issues. (2) Navigators should provide support to the patients regarding their personal organization and coordination of care. Hereby, patients’ individual situation and preferences should be comprehensively considered. (3) The provision of information and special knowledge should be a substantial part of the navigation service. This task includes referrals to organizations with specific forms of knowledge and care services. This can include, for example, medical information, disease experiences by other patients (self-help), information about mental health care.

Furthermore, it was recurrently considered important from the patients’ as well as care providers’ perspectives that the navigator should take on the role of a constant and long-term contact person for a patient and / or informal caregiver.

“For him [navigator] to tell me exactly: 'You will be discharged then. Then you can do your rehab. We will see how that all went on.' That would be a real opportunity then, then you would have a contact person. Because, well, there is no one there for you. 'No one sees me, no one hears me.' That's how I have to say that. That doesn't really work well." (Stroke, f, 69 years, diagnosis four months ago)

“As a cancer patient, everything that has to be organized is very, very difficult at first. It's so difficult because your mind is occupied with completely different things.” (Lung cancer, f, 56 years, diagnosis 4.5 years ago)

### Professional background and education of navigators

Based on the described tasks and roles of the navigator that include mostly networking activity, but also organizational and administrative tasks, a profound knowledge of existing support programs and services is considered crucial. For this reason, navigators should have a professional background rather than being volunteers. Here, we defined social work as the best-suited occupational background. Ideally, navigators should have additional experience in a health-related environment. Alternatively, persons educated in other health-related occupations like nursing care, gerontology, medical assistants or case management may be trained to fulfil the role of a navigator. Further education and training should be provided. As it will be likely that professional experience of potential navigators will vary to great extent, we propose an educational curriculum that is organized in a modular fashion. Training units include (1) introduction into patient-centered care, (2) tasks and roles of patient navigation, (3) communication skills, (4) disease-specific basic medical information and care systems, (5) knowing available services including social services, and (6) supervision. The training should be provided by integration of experts and health care providers in the respective educational modules. There are potential curricula for the education of the navigators available in Germany and the USA that may be individually added or combined with the modules of the navigation model [25, 49]. However, a standardized training for patient navigators to certify and licence navigators is not yet available and needs to be more closely researched in future studies.

### Disease-specific versus disease-transcending components

Lung cancer and stroke differ in many aspects including disease manifestation, care trajectories and health disciplines. However, needs and topics for support as described above showed a high level of overlap. Thus, the navigation model is generic and suitable for general use in the German healthcare system. The difference will lie in the specific tasks, temporality and the services the navigator will need to have knowledge about. Thus, we propose to bundle expertise of navigators according to disease types and region. This way navigators can optimally apply acquired disease-specific aspects in the care coordination for patients on the one hand and build up regional collaborative networks with involved care providers along the care trajectory on the other hand. The training modules regarding the disease, specific care systems, and available services are geared toward specific regions and diseases.

### Mode of patient-navigator interaction

Further, we discussed how the interaction between the navigator and the patient or his/her caregiver should be organized after the initial identification described above.

After contact establishment between the patient and navigator, an initial in-person meeting should be organized between the navigator and the patient. This meeting is aimed to identify the mode of further interaction that the patient prefers as well as initial support needs of the patient. In the interviews, patients wished for flexible forms of contact and interaction. This could include fixed telephone appointments, personal appointments, home visits or contact initiated by the patient on demand. In addition, the navigation should also allow for the integration and involvement of family and other informal caregivers. In this case, the navigator should further consider the needs and situation of the caregivers. Overall, the navigator should be a long-term contact person for the patient. An individual navigator should ideally be assigned to provide support to an individual patient and their caregivers along the patient’s entire care trajectory and changes in personnel should be avoided where possible to allow a long-term interaction between patient and navigator.

“Well, I think that should be clarified individually, how patients want to have that. Because they are, after all, going through highs and lows for the first time. You never know whether someone is alone or has family and is well taken care of. Well, I think you have to be flexible here." (Lung cancer, f, 57 years, diagnosis 11.5 years ago)

### Affiliation and integration of patient navigators into the health care system

The navigation model has to be associated within an existing care institution, e.g. general practice or care organizations. This way, the integration of the navigation should add no further complexity to the care trajectory by constituting yet another care element that must be accessed and organized by a patient.

Accordingly, we discussed the following procedure in the attempt to integrate the above-mentioned points: The navigator model has to be well connected within and outside the hospital setting. So that contact may be established across several venues. For example, contact with the patient or caregiver may be established by the in-house personnel like hospital social workers, nurses, or physicians. Pre-screening of patients regarding the defined target groups could be integrated in the hospital’s discharge management using questionnaires about social support and evaluation of comorbidity. Care services and physicians’ offices are other venues for the navigation model. The navigator will either contact the patient or caregiver to establish needed services and interaction form.

“But all this medical work that then has to take place afterwards [after hospital], that is, as I said, associated with legwork. That's a bit of a shit. It would be nice if all this could be done in one place. Not that you have to travel all over town to get treated somehow.” (Stroke, m, 53 years, diagnosis 2 months ago)

### Stakeholder involvement

As described above, we involved the perspective of different stakeholders that are concerned with the care of the proposed target groups in the development of the navigation model. As we aimed to develop a patient-oriented navigation model we first and foremost included the perspective of the patient and their caregivers. After the model was developed, it was presented to study participants of the interview study for final evaluation. Results showed patient agreement with the developed navigation model. However, one patient expressed concern that the model developed may not be implemented well because, in her view, it is very costly in terms of personnel.

“Sure, I can well imagine that. I mean, there are certainly people who don't want something like that, but I would like it. … He [the navigator] doesn't have to do everything for you, but he [the navigator] can definitely guide you in the right direction. I think that's a good thing. Yes sure, I can well imagine that.” (Lung cancer, m, 59 years, diagnosis 3.5 years ago)

“I think it's good that you have a contact person. Yes, who helps you then, so that's great! Yes, yes, because there are also questions. And I mean, I was lucky. But many people also have disabilities and so on. And that the social workers then know where and how to get help. Because I think there is a lot of help that is not accessed at all because people don't know that it even exists and that they are entitled to it, yes.” (Stroke, f, 72 years, diagnosis 1 year ago)

“Ok. And you can do that in terms of personnel? That will be the challenge, I think. Because, I can imagine that many patients say ‘Yes, great, come to my home.’ And then with travel and all that, it's hard in terms of personnel. But good. It's a nice approach. I hope you get that implemented.” (Lung cancer, f, 45 years, diagnosis 9 years ago)

## Discussion

In this article, we present a patient navigation model that was developed in group discussions and finalized in patient interviews based on the results of mixed methods studies that investigated barriers to care and support needs from different perspectives with special focus on the patients’ perspective. Here, we focused on two prototypic age-associated diseases, namely lung cancer and stroke, to develop a navigation model that is adjustable to different diseases with potentially very different disease trajectories.

Interestingly, our underlying qualitative results showed a high overlap between support needs for both, apparently very different, diseases (Fügemann et al., in preparation). In addition, we find that for the German healthcare system patients who may be vulnerable to receiving suboptimal care seem comparable across diseases and settings and are dependent on social support of informal care givers. Hence, we propose that the navigation model is disease transcending and we suggest a model that can be adapted to very different diseases by the described modular design in training. This is in contrast with most navigation models that are currently tested in Germany as these mostly focus on one specific disease [24, 25, 50]. In addition to the described common aspects across diseases, a navigation model must consider the disease-specific needs depending on the patient group and setting it is applied to. These needs likely differ in their quality as well as in the timing when a specific need arises [30].

Navigation models that are currently evaluated in Germany often investigate disease-specific clinical impacts of the navigation like recurrent events or rehospitalizations (e.g. Sano Project, Stroke OWL). In the international context navigation often focuses on access to care and timely care [12]. Based on the organizational and educational focus of navigation of these programs as well as on the patient-oriented focus of the here described model, we propose that effects on these clinical and often institutional outcomes may be expected to be secondary. In our patient-oriented approach we conclude that the main aim of a patient-oriented navigation in a German system is to dismantle barriers with care organization and coordination and lack of information—aspects that are important across diseases and that directly impact a patient’s personal situation. Hence, we suggest that an evaluation of a navigation assistance should primarily focus on patient-oriented outcomes like satisfaction with care, level of information or health-related quality of life.

An important question that needs to be considered is how the described patient navigation can be sustainably implemented and coordinated in the existing healthcare system and structures. The successful implementation and maintenance of navigation programs is a complex process and influenced by a variety of factors [51–53].

The described navigation will require a high number of resources upon implementation of the one-to-one support provided. This will place a high demand on a workforce from occupations that are already facing shortages like social work and nursing. Hence, sustainability requires a program to be cost effective. Further studies have shown that a navigation assistance can indeed influence the efficiency of care as measured by reducing medical costs and probably reduced number of hospitalizations [21]. However, it is important to consider the effectiveness of the model for subgroups of patients with special focus on vulnerable patient populations.

The identification of and comprehensive outreach to all vulnerable patient populations needs to be considered thoroughly for each different subgroup and setting. Limitations may occur due to the tradeoff of comprehensive outreach to the patient and the timing, when the patient is in need of a navigation assistance. In the case of stroke patients, a stroke unit or hospital setting may be a location where all patients can be reached shortly after a stroke event. However, in this case patients may be reached too early along their care trajectory, as support needs for a navigation assistance may not be abundant for a patient shortly after the stroke incident and they may not accept the offer of a navigation assistance at this time point. Nevertheless, problems and questions for patients may arise later when they are at home and face limitations. That said, focusing identification to settings like rehabilitation hospitals or specialized practices limits the patient population and vulnerable patients as not all patients will get or use these health care services [54]. In addition, finding and organizing a specialist appointment is a described barrier for the patient and a task for the navigator per se. Hence, we propose a high level of flexibility where different settings for the contact and identification of the patient should be used and the active contact initiated by the patients or their caregivers should be possible.

Overall, the navigator should reach or be reachable by all patients in need. Further, he/she should build a bridge between all people involved in the patient’s care. Of note, these complex issues need to be flexibly tailored to a patient population or care setting in a way that does not add a further level of complexity to the care paths of the patient. The specific point of implementation should hence be flexible regarding the setting it is imbedded in and to the patient group at which it is directed. In addition, the recruitment of caregivers seems essential, as they are often the main organizers of care. And as in the case of stroke, a substantial number of patients are not able to give informed consent [55] and it appears necessary to include caregivers to reach also highly affected patients for navigation [56]. These are important issues as it is reported for health care interventions that acceptance of a healthcare offer by patients can be impaired by factors like form of provider or location [57]. However, the above described issues of sustainability and implementation of a navigation program are complex and necessitate investigation and evaluation in the real-life healthcare system. To address some of these issues a subsequent feasibility study to evaluate a navigation intervention based on the described model will be conducted.

Our results further indicate that there are several support resources in Germany beyond the medical-therapeutic treatment of a patient including counselling services, self-help groups or special athletic programs, but these resources are often not well known neither to patients nor to health care providers [7, 9]. The navigator’s task will be to build a bridge between these services, medical care providers and the patient. It is possible that not only the patients directly benefit from the work of a navigator, it may also have an additional effect by increasing the knowledge about programs and services in a region and hence have an integrating effect on all parties concerned with the care of patients [23]. Higher levels of support with care coordination and with open questions provided by a navigator may even positively affect the interaction with patients by decreasing the high burden of organizational tasks experienced from the physician’s perspective [41].

## Data Availability

Research results and data of the research components of the mixed-methods studies, that where basis of the Delphi-adapted process, were published separately as indicated throughout the manuscript. Further relevant data analyzed during the current study are included in the article. Due to data protection restrictions, additional data are not available.
